# ODNA: a manually curated database of noncoding RNAs associated with orthopedics

**DOI:** 10.1093/database/baz126

**Published:** 2019-11-28

**Authors:** Changcheng You, Kai Zhu, Qiuhua Zhang, Jnglong Yan, Yufu Wang, Jing Li

**Affiliations:** 1 Department of Orthopedic Surgery, Second Affiliated Hospital, Harbin Medical University, Harbin, China; 2 Department of Pathology and Centre of Electron Microscope, Faculty of Basic Science, Harbin Medical University, Harbin, China; 3 Harbin Children’s Hospital, Harbin, China; 4 Laboratory Medicine and Pathology, Faculty of Medicine & Dentistry, University of Alberta, Edmonton, Canada

## Introduction

Orthopedics is an important branch of medicine concerned with disorders of the bones, joints, spinal cord, peripheral nerves, muscles, connective tissue and cartilage (1). Orthopedic disorders, such as back pain and arthritis, have high incidence rates and affect the quality of life of most people at some point during their lifetime (2,3). In addition, many disorders of other body systems, including the vascular, nervous, immune and metabolic systems, may also affect the musculoskeletal system and complicate the diagnosis and treatment of orthopedic diseases (4–6). The causes of diseases involving the musculoskeletal system have not been well elucidated. Currently, multiple mechanisms, including proteomic, transcriptomic, genomic, epigenetic regulatory and environmental mechanisms, have been shown to be involved in the development of orthopedic disorders (7,8).

Over the past decade, studies have revealed new classes of RNA molecules, including microRNAs (miRNAs), small nucleolar RNAs (snoRNAs), long noncoding RNAs (lncRNAs), PIWI-interacting RNAs (piRNAs) and circular RNAs (circRNAs). These noncoding RNAs (ncRNAs) not only regulate a variety of cell functions in the development of and physical processes involving skeletal muscle but also contribute to a number of orthopedic-related disorders (9–11). For example, miRNAs are believed to regulate the differentiation of osteoblasts and osteoclasts through upregulation or downregulation or sponging (12). LncRNAs have been identified as critical molecules that regulate bone and cartilage degeneration, promote bone metastases and repair spinal cord injuries. It has also been reported that the abnormal expression of lncRNAs leads to the malformation of bones and homeotic transformation (13). Differentially expressed circRNAs may play important roles in the differentiation and proliferation of stem cells. The abnormal expression of circRNAs is also involved in arthritis, osteoporosis and hereditary bone diseases (14–16). In recent years, studies on the associations between ncRNAs and orthopedic disorders have rapidly accumulated. However, the data about these associations have remained scattered.

To facilitate investigations into the associations between orthopedic disorders and ncRNAs, we constructed a public database, the Orthopedic Diseases ncRNAome Atlas (ODNA), which contains manually curated data on orthopedic disease-associated, experimentally validated ncRNAs.

## Materials and methods

We used Medical Subject Headings (MeSH) terminology to collect the disease names. To construct a high-quality database, we referred to the steps used by published databases such as NSDNA (17), Lnc2Cancer (18) and the Coral Traits Database (19). The ncRNA information was obtained from miRBase (20), NONCODE (21), lncRNAdb (22), snoRNA-LBME-db (23) and piRNABank (24).

**Figure 1:**
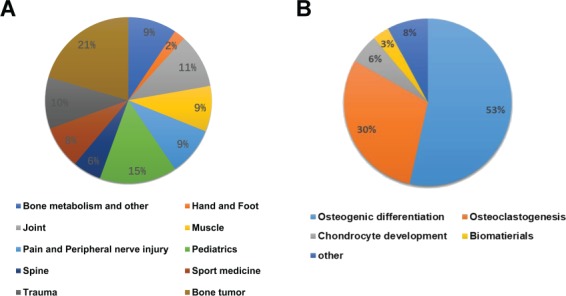
Descriptive statistics regarding the database. (**A**) Distribution of diseases in the ODNA. (**B**) Distribution of the physical processes regulated by the ncRNAs in the ODNA.

In the process of obtaining the information, the following five steps were applied: the collection of the names of associated diseases and the ncRNA category, identification of relevant articles, exclusion of noneligible articles, extraction of useful data from the eligible articles and validation of the data by the third author. Electronic searches were performed independently by two reviewers in PubMed. The following were used as either keywords or MeSH terms in all combinations for the search: `each ncRNA symbol/ncRNA category name’ and `each orthopedic disease name/physical process’. The criteria for including ncRNAs in ODNA were as follows: (1) the involved disease was related to the musculoskeletal system; (2) the ncRNA was experimentally validated and has abnormal expression in subjects with the involved disease (statistically P > 0.05); (3) the samples and species of the samples used in the validation were clearly stated; and (4) the experimental methods used to analyze the ncRNA analysis were clearly described. The exclusion criterion was repeated use of the same data.

We downloaded all published studies and available supplementary files describing the associations between the diseases/physical processes and ncRNAs. Two authors independently extracted the data from the articles. We collected the following detailed information from the published studies: disease and ncRNA name, species, ncRNA expression pattern, experimental methods, target information, samples used, description of ncRNA function and references. The data were divided into data derived from high-throughput and low-throughput experiments, as previously described (17). High-throughput methods included microarray and next-generation sequencing, whereas low-throughput methods included real-time polymerase chain reaction, northern blotting, western blotting and luciferase reporter assays. Additionally, the public accession number and chromosomal location of each ncRNA are also provided on each results page.

The accuracy of the data entered was confirmed by a third reviewer. The retrieved results were last updated in February 2018. ODNA was built using MySQL and JSP. The scripts were written in Java. The web service is running on an Apache Tomcat web server.

## Results

**Figure 2:**
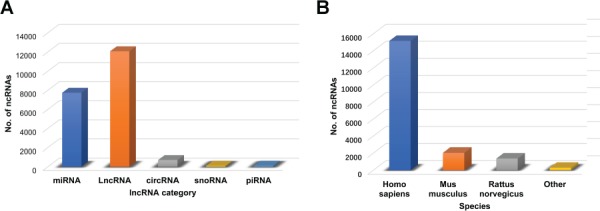
Descriptive statistics regarding the ncRNAs in the database. (**A**) Numbers of RNAs in the different RNA categories. (**B**) Numbers of RNAs in the various species.

**Table 1:** Statistics for the ncRNA entries in the ODNA database

Topic	miRNA	lncRNA	snoRNA	circRNA	piRNA	Publications (n)
Category of Diseases						
Tumor	1763	1416	34	16	30	260
Bone metabolism/Other	613	80	9	N/A	N/A	89
Pediatrics	251	10	5	1	N/A	60
Sport medicine	131	66	6	N/A	N/A	21
Trauma	401	69	N/A	N/A	N/A	47
Joint	700	5476	58	213	4	198
Spine	227	3270	N/A	636	N/A	21
Pain/Peripheral nerve	710	474	7	83	18	73
Muscle	130	N/A	N/A	N/A	N/A	21
Hand and foot	154	N/A	N/A	N/A	N/A	4
Physical process	1127	2530	N/A	62	18	89

**Figure 3:**
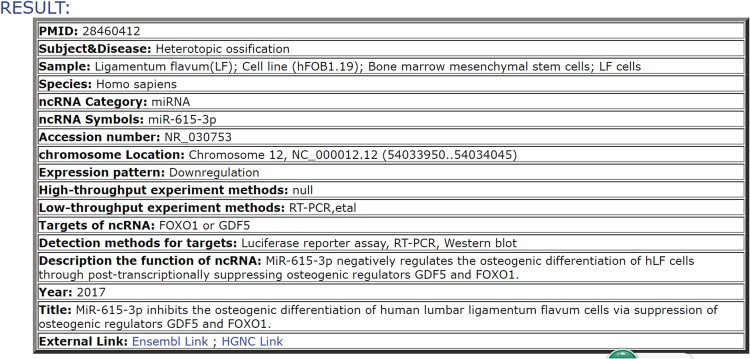
Results table containing the core information included in the database. In this example, we report the result for an ncRNA.

The database is available at http://bio-bigdata.hrbmu.edu.cn/ODNA. The current ODNA database consists of 6207 miRNAs, 13 391 lncRNAs, 1011 circRNAs, 119 snoRNAs and 70 piRNAs and documents 175 orthopedic diseases and 62 physical processes across 11 species. The ncRNAs were curated from over 700 articles obtained from PubMed. The datasets in this article are available from the ODNA (http://bio-bigdata.hrbmu.edu.cn/ODNA/download.jsp) and Figshare repository record (https://figshare.com/s/519d4a09fa4e46feff99). The diseases and physical processes regulated by the included ncRNAs are displayed in Figure 1. Statistics regarding the ncRNAs in the ODNA are displayed in Figure 2. Tables 1 and 2 show the statistics regarding the ncRNA entries in the ODNA database. Each entry contains the following detailed information: the ncRNA name, the species, the sample used for detection, the associated disease name/physical process (subject), the expression pattern (upregulation or downregulation) of the ncRNA, the high-throughput or low-throughput methods used to investigate the expression pattern, the targets of the ncRNA and the corresponding experimental methods, a brief description of the function of the ncRNA and the references [PubMed ID (PMID) and publication year]. We also provided several links to web-based tools, which allow users to identify more specific information of an ncRNA. A results table is used to show the details for each ncRNA; the results table can be displayed by selecting the specific RNA of interest (Figure 3). The data obtained were extensively checked for double entries, errors and inconsistencies. Data on the disease name/physical process, ncRNA symbols, regulation pattern, experimental methods and function were all confirmed by two reviewers. In the database, the references and PMIDs are provided for each entry, enabling any user to directly obtain access to the original articles.

**Table 2:** Disease and physical process included in the ODNA

Category	Disease name/physical processes included
Diseases	
Tumor	Osteosarcoma, Ewing sarcoma, chordoma, bone metastasis, chondrosarcoma, synovial sarcoma, MPNST, rhabdomyosarcoma, etc.
Bone metabolism/other diseases	Osteoporosis, osteomyelitis, exotic ossification, AS, DTV, heterotopic ossification, acute graft-versus-host disease, etc
Pediatrics	DMD, spinal bifida, BMD, obstetric brachial plexopathy, pediatric abuse, neural tube defect, osteogenesis imperfecta, clubfoot, etc.
Sport medicine	ACL injury, meniscal injury, rotator cuff tear, glenohumeral arthritis, Achilles tendon injury, etc.
Trauma	Bone fracture, spinal cord injury, brachial plexus avulsion injuries, post-traumatic stress, osteoporotic fracture, hindlimb ischemia, etc.
Joint	Osteoarthritis, RA, osteonecrosis of the femoral head, lyme arthritis, gouty arthritis, juvenile idiopathic arthritis, psoriatic arthritis, etc.
Spine	Intervertebral disc degeneration, adolescent idiopathic scoliosis, OPLL, osteoarthritis of spine, etc.
Pain/peripheral nerve	Neuropathic pain, peripheral nerve injury, cubital tunnel syndrome, bone cancer pain, chronic low back pain, age-related tendon degeneration, etc.
Muscle	Muscular dystrophy, exercise muscle injury, muscle contusion, myositis, etc.
Hand and foot	Dupuytren’s disease, diabetic foot, amputated fingers, etc.
Physical process	Osteogenic differentiation/proliferation, chondrogenic differentiation, osteoclastogenesis, etc.

ACL, anterior cruciate ligament; AS, ankylosing spondylitis; BMD, Becker muscular dystrophy; DMD, Duchenne muscular dystrophy; DVT, deep vein thrombosis; MPNST, malignant peripheral nerve sheath tumor; OPLL, ossification of the posterior longitudinal ligament; RA, rheumatoid arthritis.

**Figure 4:**
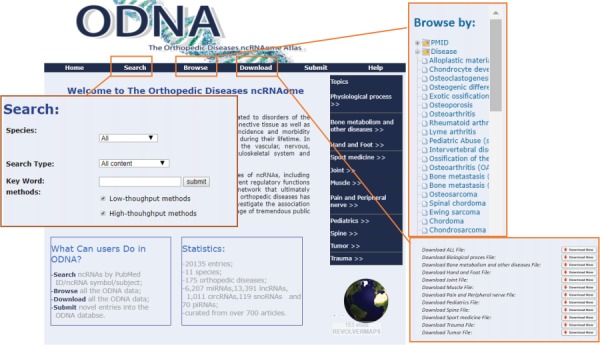
A schematic workflow of the ODNA.

The ODNA provides a user-friendly web interface that facilitates easy database querying (Figure 4). On the `Search’ page, the ODNA allows users to search for miRNAs, lncRNAs, siRNAs, snoRNAs, piRNAs, disease/physical processes and PMIDs. Additionally, the ODNA enables users to select specific species from the species pull-down menu. On the `Browse’ page, users can browse miRNAs, lncRNAs, siRNAs, snoRNAs, piRNAs, diseases, species and PMIDs. When a particular node is clicked, the corresponding results are displayed.

Researchers making use of the database are encouraged to assess the validity and accuracy of the data and send us feedback through the website interface. The data will be checked and updated by our team. After verification, users will be able to upload data to the database. The ncRNA symbol, regulation pattern and reference title or PMID are the required fields. In the future, the intention is for the information for any given entry in the database to be curated on a voluntary basis.

## Discussion

In recent years, with the rapid accumulation of ncRNA studies, several ncRNA-associated databases, such as miR2Disease (25), NSDNA (17), LncRNADisease (26) and HMDD (27), were constructed to provide tools for ncRNA research. However, a database of ncRNAs associated with the musculoskeletal system was still lacking. Therefore, the current version of ODNA may serve as a tool for researchers investigating the functions of ncRNAs, contributing to the understanding of the pathological mechanisms underlying orthopedic diseases.

Since the first profiling studies of ncRNAs during the stages of osteoblast and osteoclast differentiation, an increasing number of publications related to bone biology and pathologies have been published, as have many comprehensive reviews summarizing the characteristics of ncRNAs in skeletal cells (9,16). A large number of ncRNA and orthopedic disease studies demonstrated that the multifaceted components of the different classes of ncRNAs contribute to the epigenetic control of the skeleton and skeletal diseases far more than do DNA mutations (28,29). However, a high-quality and well-curated public resource specifically for orthopedic disease–ncRNA association data has thus far been unavailable. Therefore, we decided to construct a platform to integrate this valuable information and provide a way to acquire novel knowledge with big data mining (9). The ODNA database includes not only data sources and a user-friendly interface but also useful toolkits to provide a one-stop service for researchers. With the help of the ODNA, users can retrieve the expression pattern and function of an ncRNA, which contributes to the understanding of the precise roles of ncRNAs in disease development or physical processes. Moreover, ncRNAs and the protein machineries that are involved in their biogenesis or activities have become the targets of novel therapeutic approaches (30–32). The integrated information drawn from the literature, such as the targets of ncRNAs, can be used when developing ncRNA-based therapies for orthopedic-related diseases.

To the best of our knowledge, the ODNA is the first database focusing on orthopedic-associated ncRNAs. One strength of this database is that it includes almost all known orthopedic diseases, including arthritis, bone tumor/metastasis, spinal diseases, metabolic diseases, trauma, pediatric congenital malformations, muscle diseases, pain and sport-related injuries. In contrast, several published databases, such as LncRNADisease and miR2Disease, only document a limited number of orthopedic-related ncRNAs and only include one ncRNA category. Moreover, the ODNA includes the circRNAs associated with the musculoskeletal system, while the above-mentioned databases do not include circRNA-related data.

Previously, most disease-related databases focused on the pathological mechanisms underlying disease progression, and the data regarding the physical functions of the ncRNAs were not well documented. Therefore, another strength of this database is that it includes data pertaining to the physiological processes of musculoskeletal cells, including the proliferation of bone cells, differentiation of stem cells, regeneration of nerve cells/muscular cells and development of the skeleton. As an integral part of orthopedics, biomaterials/implants influence the levels of ncRNAs, and this information is also included in the section on physical processes in the ODNA. Implants are always fixed to bone, but the mechanisms by which these biomaterials act on bone are unknown. Therefore, the ncRNA data related to implants may contribute to the understanding of the molecular mechanism by which implants affect bone regeneration.

In the ODNA, the majority of the entries are associated with miRNAs and lncRNAs, suggesting that these categories of ncRNAs play key roles in the diagnosis and treatment of orthopedic-related diseases. The number of miRNA- and lncRNA-related studies has been steadily increasing over the past 10 years (33). These experimental reports have revealed that miRNAs and lncRNAs contribute to almost every step of osteogenesis and bone homeostasis and have identified that they are potential novel diagnostic biomarkers and drug targets for bone diseases (34). Several drugs used to treat orthopedic-related diseases by targeting ncRNAs have demonstrated substantial progress in preclinical and clinical studies (35). Notably, the number of circRNA-related publications has increased significantly in recent years. Studies have shown that circRNAs function as ceRNAs or miRNA sponges and translate proteins, interact with RNA-binding proteins, regulate the stability of mRNAs and modulate gene transcription (36). CircRNAs can serve as novel therapeutic targets and noninvasive biomarkers in bone diseases, including arthritis, osteoporosis and malignancies (37–39). With the development of high-throughput technologies, circRNAs have become another hot topic in the field of orthopedics.

In summary, the ODNA is the first manually curated database of ncRNAs associated with the field of orthopedics, and it is a valuable resource for researchers seeking to understand the pathological mechanisms underlying musculoskeletal diseases. We hope the current version of the database and future updates will provide a context for the diagnosis and treatment of orthopedic-related disorders and can be used by different research communities.
